# Effects of Whole Body Electrostimulation Associated With Body Weight Training on Functional Capacity and Body Composition in Inactive Older People

**DOI:** 10.3389/fphys.2021.638936

**Published:** 2021-04-01

**Authors:** Alexandre Lopes Evangelista, Angelica Castilho Alonso, Raphael M. Ritti-Dias, Bruna Massaroto Barros, Cleison Rodrigues de Souza, Tiago Volpi Braz, Danilo Sales Bocalini, Julia Maria D'andréa Greve

**Affiliations:** ^1^Laboratório de Fisiologia e Bioquímica Experimental, Centro de Educação Física e Esporte, Universidade Federal do Espirito Santo, Vitoria, Brazil; ^2^Programa de Mestrado Ciências do Envelhecimento, Universidade São Judas Tadeu, São Paulo, Brazil; ^3^Programa de Pós-graduação em ciências da reabilitação, Universidade Nove de Julho, São Paulo, Brazil; ^4^Laboratório de Avaliação do Movimento Humano, Universidade Metodista de Piracicaba, Piracicaba, Brazil; ^5^Departamento de Ortopedia e Traumatologia, Universidade de São Paulo Faculdade de Medicina, São Paulo, Brazil

**Keywords:** older adults, electrostimulation, physical function, body composition, functional fitness

## Abstract

**Objective:** To analyze the effects of whole body electrostimulation (WB-EMS) with body weight training on functional fitness and body composition of older men.

**Methods:** Twenty physically inactive older men were randomized into: Control group (control), performed the body weight exercise training wearing electrostimulation clothing, but without receiving electrical current stimuli (*n* = 10), and body weight associated with whole body electrostimulation group (BW+WB-EMS), performed the body weight exercise training wearing electrostimulation clothing plus whole body electrostimulation (*n* = 10). The training sessions were performed twice a week for 6 weeks and included eight exercises using body weight, performed in two sets of eight repetitions. Physical function was assessed using a battery composed of seven tests, six derived from the Senior fitness test and a handgrip strength test. We also measured the muscle thickness (MT) of the biceps and triceps brachii and vastus lateralis.

**Results:** The BW+WB-EMS group presented increased (*p* < 0.05) performance in the 30-s chair stand test (10.2 ± 3.3 vs. 13.8 ± 5.0 reps), arm curl (16.6 ± 3.9 vs. 19.9 ± 6.1 reps), 6-min walk test (402 ± 96 vs. 500 ± 104 m), and handgrip strength test (30 ± 11 vs. 32 ± 11 kgf). The BW+WB-EMS group also presented increased MT (*p* < 0.05) in the biceps brachii (17.7 ± 3.0 vs. 21.4 ± 3.4 mm), triceps brachial (14.7 ± 3.6 vs. 17.5 ± 4.1 mm), and vastus lateralis muscles (15.1 ± 2.6 vs. 18.6 ± 4.3 mm). Moderate correlations were found in arm curl (*p* = 0.011, *r* = 0.552) but not handgrip strength (*p* = 0.053, *r* = 0.439) with changes in the biceps MT. Moderate changes in the 6-min walk distance were significantly correlated with changes in vastus lateralis MT (*p* = 0.036, *r* = 0.471). There was a moderate correlation between the changes in the 30-s chair stand test (*p* = 0.006, *r* = 0.589) and changes in the vastus lateralis MT. Furthermore, although a moderate correlation (*r* = 0.438) was found between triceps MT and handgrip strength no significant difference (*p* = 0.053) was reported. Additionally, there were no statistical differences in any parameters for the control group.

**Conclusion:** WB-EMS with body weight training increased functional fitness and MT in physically inactive older men.

## Introduction

Aging is associated with loss of muscle strength and muscle atrophy (Aoyagi and Shephard, 1992), which contributes to functional decline. Resistance exercise using body weight has been shown to improve strength, muscle mass, and physical function in older subjects (Van Roie et al., 2013). However, following the recommendations for the practice of resistance exercise can be difficult and only the minority of older people attain the minimum doses necessary to obtain the aforementioned benefits (Carlson et al., 2010). The reasons frequently given for not exercising are time limitations, physical limitations, or little enthusiasm to perform exercise alone (Kemmler et al., 2017).

In order to improve motivation to practice physical exercises (Fortier et al., 2016), as well as to maximize the results of more traditional interventions, several strategies have been developed. Among them, training with whole body electrostimulation (WB-EMS) has gained popularity and is increasingly offered by gyms and fitness centers in many countries, including Brazil (Evangelista et al., 2019b). Whole body electrostimulation has been shown to potentiate the effects of resistance training performed with body weight, leading to greater increases in strength and muscle mass (Kemmler et al., 2018). To the best of our knowledge few studies (Filipovic et al., 2012; Kemmler et al., 2018; Evangelista et al., 2019b) have investigated strength gains (Filipovic et al., 2012; Kemmler et al., 2018; Evangelista et al., 2019b), functional capacity (Jee, 2018), muscle hypertrophy (Kemmler et al., 2018; Evangelista et al., 2019b), and body composition (Kemmler et al., 2018). One hypothesis to explain these outcomes could relate to the increase in mechanical stress, which has been postulated as one of the main stimuli for the process of myofibrillar protein synthesis and consequent muscle hypertrophy. In addition, the possible additional recruitment of muscle fibers resulting from the WB-EMS can maximize energy expenditure and metabolic stress. In this case, metabolic stress has been noted as one factor that contributes to increases in the cross-sectional area of the muscle. In addition, improvements in performance (Filipovic et al., 2012), psychological profile (Jee, 2018), and reduction in pain in day-to-day activities have also been described after exercise with this device (Kemmler et al., 2018; Weissenfels et al., 2019). Whole body electrostimulation is a time-efficient, joint-friendly, and highly individualized exercise technology making it a good choice for older subjects (Brigatto et al., 2019).

Kemmler et al. (2018) studying the effects of combined WB-EMS and protein supplementation on local and overall muscle and fat distribution in older men with sarcopenic obesity showed that 1.5 sessions of 20 min/week for 16 weeks increased muscle- and maintained fat-volume after WB-EMS training. Additionally, changes in gait velocity, leg-extensor strength, and advanced lower extremity function after WB-EMS. Demonstrating that WB-EMS is a highly customizable option for people either unable or unmotivated to conduct intense (resistance) exercise protocols.

Despite the benefits, studies on the effects of WB-EMS in older subjects are scarce. Thus, the aim of the current work is to analyze the effects of WB-EMS associated with body weight training on the functional capacity and body composition of older people. We hypothesized that WB-EMS with body weight training would promote improvements in functional parameters and muscle mass, compared to the group that did not receive electrical current stimuli.

## Methods

### Subjects

Thirty-six sedentary older men were initially invited and volunteered to participate in this study. Volunteers were selected through the analysis of medical records and brief phone contact to explain the procedures. The older men who agreed to participate in the study visited the laboratory, where all the procedures were explained in detail, doubts clarified, and the volunteers read and signed an informed consent. All the procedures were in accordance with the ethical standards of the responsible committee on human experimentation of Anhembi Morumbi University (n° 4.028.844/2020) and with the Helsinki Declaration.

After applying the exclusion criteria: participating in a regular and structured physical activity program in the 6 months prior to the study; recent hospitalization; motor deficiency; symptomatic cardiorespiratory disease; non-controlled hypertension or metabolic syndrome; severe renal or hepatic disease; cognitive impairment or debilitating conditions; marked obesity with inability to exercise; recent bone fracture (during the previous 2 years), six volunteers were excluded.

**Figure 1:**
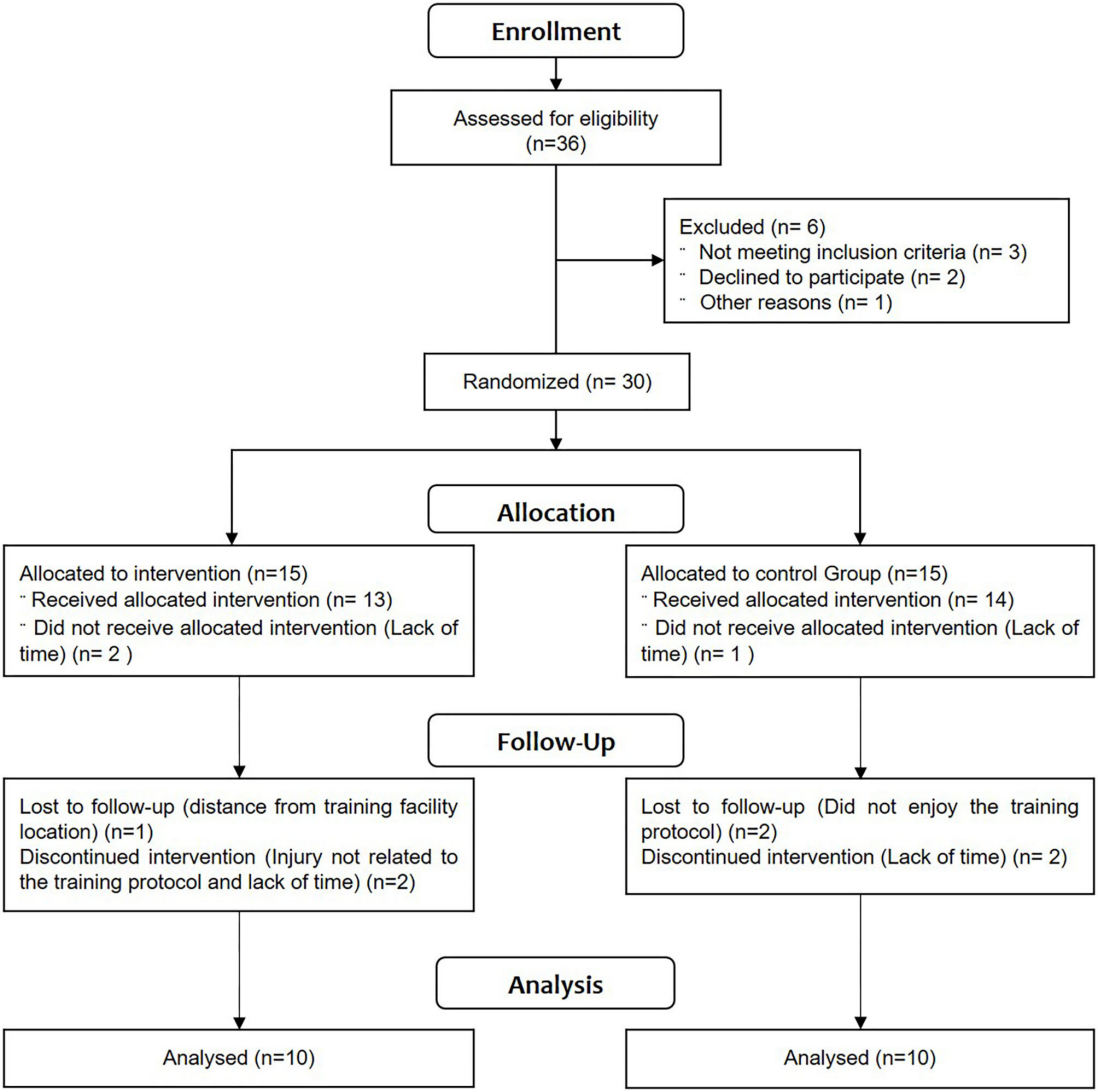
Diagram illustrating participant flow through experimental study design.

All participants (30 subjects) were then assigned to either the body weight associated with whole body electrostimulation group (BW+WB-EMS) or control group using a computerized random-number generator. The randomization process occurred in blocks of five subjects. Each block resulted in the allocation of two subjects to the BW+WB-EMS and two subjects to the control group, ensuring a recruitment balance of 1:1 throughout the study. Both groups performed the same exercise protocol with body weight, however the BW+WB-EMS group (*n* = 15) performed the exercises with whole body electrostimulation, while the control group (*n* = 15) carried out the exercises without receiving electrical current stimuli. During follow up, three subjects in the BW+WB-EMS group and four in the control group dropped out of the study. Thus, 20 subjects (mean age 75.1± 6.58 years) were analyzed in this study, as shown in Figure 1.

### Procedures

The exercise interventions consisted of 6 weeks of training performed twice weekly. For both groups, the training sessions included a 5-min warm-up followed by eight exercises using body weight, with two sets each and eight repetitions per set. The following exercises were applied: standing fly, squat, neutral pull, standing unilateral knee flexion, lateral raise, simultaneous curl, simultaneous elbow extension, and standing abdominal. The cadence of repetitions was conducted in a controlled fashion, with concentric and eccentric actions of approximately 2 and 4 s, respectively, with a total repetition duration of approximately 6 s. Subjects in the control group were asked to contract the muscle at maximum intensity through the full range of motion without the use of external load. The subjects in the BW+WB-EMS group were asked to rate the average intensity of the exercise session and the regional intensity of the EMS on a rating of perceived exertion scale, and the intensity was maintained between 7 and 8 throughout the experimental protocol (Evangelista et al., 2019b). Research staff, certified in the use of the technology, supervised all training sessions, provided verbal encouragement, and ensured that the subjects performed the correct number of sets and repetitions with the correct exercise technique.

All subjects completed two familiarization sessions separated by a minimum of 72 h before beginning the experimental protocol; both sessions occurred 1 week after the maximum dynamic strength test, functional tests, and muscle thickness (MT) assessments. During these sessions, subjects were familiarized with the exercises and proper techniques.

**Figure 2:**
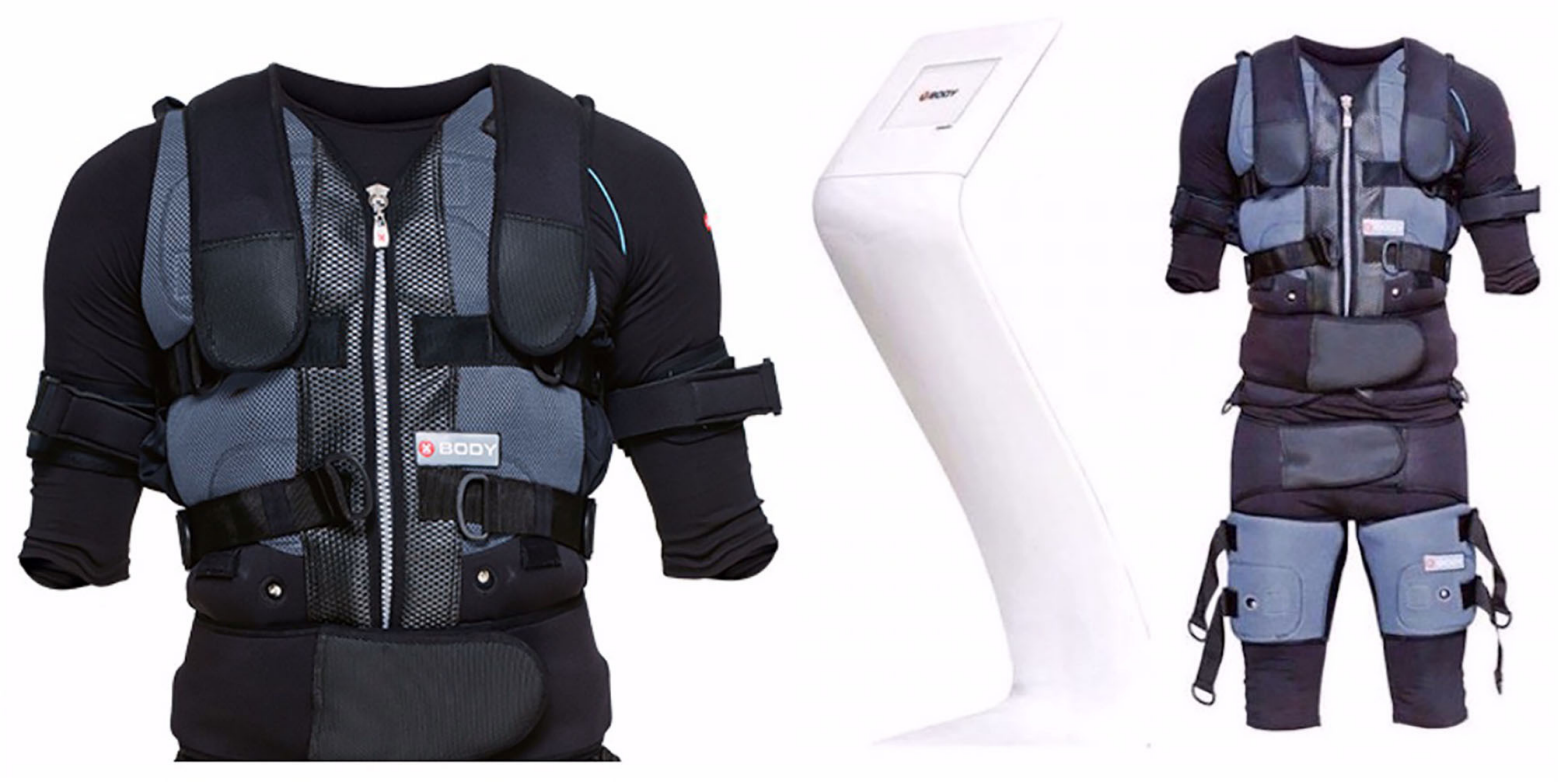
X-body Whole body electrostimulation equipment.

In the BW+WB-EMS group, the XBody (Dorsten, Nordrhein-Westfalen, Germany) equipment (Figure 2) was adjusted to release a bipolar electrical current with a frequency of 85 Hz, pulse amplitude of 350 μs, intermittent, with 4 s of direct pulse stimulation, and 2 s of replacement (Table 1). In the control group, although the subjects wore the device, it remained turned off throughout the session.

**Table 1:** Whole body electrostimulation protocol design.

**Program variables**	**Stimulation**
Stimulation frequency	85 Hz
Impulse duration	4 s
Impulse break	2 s
Pulse breadth	350 μs
Impulse type	Bipolar
Duration	~20 min
Regional intensity (Borg CR-10 scale)	7–8

Subjects were asked to refrain from performing any type of additional exercise regimen during the study. In addition, participants were advised not to change their dietary intake/usual nutrition during the study. Both groups received general guidance on healthy eating habits at the beginning of the study.

### Evaluated Parameters

#### Body Composition

Body composition analysis included full body bioimpedance analysis (BIA) and evaluations of lower and upper limb MT. For the bioimpedance assessment, the InBody_230_ device was used. Prior to testing, participants were required to abstain from exercise and alcohol or excessive caffeine consumption for at least 72 h and food and drink for 4 h, except for water which could be consumed until 45 min before testing. The parameters obtained by the device were: weight (Kg), height (cm), body mass index (Kg/m^2^), lean mass (Kg), and %fat (%).

#### Muscle Thickness

The MT of the biceps brachii, triceps brachii, and vastus lateralis was assessed using an ultrasound-imaging unit (Mindray; DP10; Shenzhen, China) with a wave frequency of 7.5–10 MHz. The MT assessment was conducted following previous procedures by Brigatto et al. (2019), with the ultrasound probe applied perpendicularly to the skin for measurement. Water-soluble gel was used on the transducer to aid acoustic coupling and remove the need for excess contact pressure on the skin. Muscle thickness was defined as the distance from the interface of the muscle tissue and subcutaneous fat to the bones. Imaging was performed on the right side of each subject's body. The subjects were required to fast for 3 h before testing, and MT assessments were performed at the same time of day at pre- and post-testing. The biceps and triceps MT assessments were performed at 60% distal between the lateral epicondyle of the humerus and the acromion process of the scapula, and for the vastus lateralis, assessments were performed at 50% distal between the lateral condyle of the femur and greater trochanter. The test–retest ICCs from our laboratory for the biceps, triceps, and vastus lateralis were 0.996, 0.998, and 0.999, respectively and the standard errors of the means were 0.29, 0.42, and 0.41 mm, respectively.

#### Functional Fitness

The primary outcome of this study was physical function assessed by a battery composed of seven tests, six tests derived from the Senior fitness test, and the handgrip strength test. These tests evaluate different components of physical function, such as strength of upper and lower limbs, power, flexibility, balance, agility, speed, and cardiorespiratory function.

The 30-s chair stand test, arm curl, 6-min walk test, 2 min step test, back scratch test, and 8 feet Up-and-Go test were performed following the procedures previously described by Rikli and Jones (1999). The handgrip strength test was performed using a hydraulic hand dynamometer (SAEHAN SH5001®), previously calibrated, and following the procedures described by the American Society of Hand Therapists (Fess, 2002).

#### Statistical Analyses

The normality and homogeneity of the variances were verified using the Shapiro-Wilk and Levene tests, respectively. Prior to analysis, all data were log-transformed for analysis to reduce bias arising from non-uniformity error (heteroscedasticity). The mean, standard deviation (SD), and 90 and 95% confidence intervals (CI) were used after data normality had been verified. To compare mean values of the baseline descriptive parameters between-groups (CON vs. BW+WB-EMS), an unpaired *t*-test was used. A mixed ANOVA with time as within-subjects factor and group as between-subjects factor was used to compare time effect (pre vs. post) × two groups (CON vs. BW+WB-EMS) for the variables body mass, %Fat, lean mass, MT_BB_, MT_TB_, MT_VL_, sitting-rising test, arm curl, stationary march test, back scratch test, 8 feet Up-and-Go, 6-min walk test, and isometric handgrip strength. *Post-hoc* comparisons were performed with the Bonferroni correction. Assumptions of sphericity were evaluated using Mauchly's test. When sphericity was violated (*p* < 0.05), the Greenhouse–Geisser correction factor was applied. Data normality was confirmed by Shapiro Wilk's test. Pearson correlations were performed and classified as; weak (*r* between 0.20 and 0.39); moderate (*r* = 0.40 to 0.59); moderate to strong (*r* = 0.60 to 0.79); and strong (*r* > 0.80). In addition, effect sizes were evaluated using a partial eta squared (η^2^_*p*_), with <0.06, 0.06–0.14, and >0.14 indicating a small, medium, and large effect, respectively. All analyses were conducted in SPSS-22.0 software (IBM Corp., Armonk, NY, USA). The adopted significance was *P* ≤ 0.05.

## Results

As presented in Table 2, significant differences were found in baseline parameters between the control group and the BW+WB-EMS group for the stationary march test and the Back scratch test. The values of sitting-rising test, arm curl, 6-min walk test, and handgrip strength were different from the pre-intervention value and the control group. However, the values in the stationary march test, and back scratch test, left and right side, were different only for the control group. No differences were found in the 8 feet up-and-go test.

**Table 2:** Alterations on body composition and functional fitness after 6 weeks of strength training combined with electrical muscle stimulation.

**Parameters**	**Pre**	**Post**	**Δ%**	**MD [95%CI]**	**Time**	Time*Group
					***p*-value**	***p*-value**
**BODY COMPOSITION**						
**Body mass (kg)**						
Control	69.9 ± 11.7	67.6 ± 11.9	0.5	2.3 [−1.2 to 2.8]	= 0.402	= 0.507
BW+WB-EMS	76.2 ± 16.2	76.2 ± 16.9	−0.6	−0.1 [2.2 to 5.4]	= 0.504	
**Fat body (%)**						
Control	31.8 ± 12.2	31.8 ± 12.7	−0.1	0.1 [−0.7 to 0.9]	= 0.672	= 0.534
BW+WB-EMS	34.6 ± 6.6	35.0 ± 7.1	1.0	0.4 [−0.5 to 2.5]	= 0.388	
**Lean mass (kg)**						
Control	45.7 ± 8.6	45.6 ± 8.2	−0.2	−0.1 [−0.4 to 0.2]	= 0.409	= 0.438
BW+WB-EMS	49.4 ± 12.1	50.0 ± 11.1	1.1	0.6 [−0.3 to 1.5]	= 0.327	
**FUNCTIONAL FITNESS**						
**Sitting-rising test (reps)**						
Control	11.8 ± 4.9	12.0 ± 2.7	1.7	0.2 [−0.1 to 0.5]	= 0.192	= 0.024
BW+WB-EMS	10.2 ± 3.3	13.8 ± 5.0^†^ ^#^	35.3	2.6 [1.3 to 3.9]	= 0.022	
**Arm curl (reps)**						
Control	14.3 ± 3.2	14.5 ± 2.9	1.4	0.2 [−0.8 to 1.2]	= 0.289	= 0.012
BW+WB-EMS	16.6 ± 3.9	19.9 ± 6.1^†^ ^#^	19.9	3.3 [0.9 to 5.7]	= 0.007	
**Stationary march test (reps)**						
Control	36.8 ± 11.4	37.4 ± 9.2	1.6	0.8 [−0.4 to 2.0]	= 0.289	= 0.045
BW+WB-EMS	51.2 ± 23.8^#^	52.5 ± 19.0^#^	2.5	1.3 [0.1 to 2.5]	= 0.183	
**Back scratch test-left (cm)**						
Control	19.0 ± 16.1	18.5 ± 15.2	−2.7	−0.5 [−2.1 to 1.1]	= 0.128	= 0.023
BW+WB-EMS	9.1 ± 11.1^#^	9.5 ± 8.3^#^	4.4	0.4 [−0.7 to 1.5]	= 0.107	
**Back scratch test-right (cm)**						
Control	16.4 ± 13.9	15.0 ± 12.5	−8.4	−1.4 [−3.6 to 0.8]	= 0.338	= 0.042
BW+WB-EMS	7.0 ± 8.5^#^	5.1 ± 7.0^#^	−27.1	−1.9 [−3.9 to 0.1]	= 0.256	
**8 feet up-and-go (s)**						
Control	10.5 ± 3.3	9.4 ± 3.0	−10.7	−1.1 [−3.6 to 1.4]	= 0.202	= 0.132
BW+WB-EMS	8.6 ± 3.0	7.2 ± 2.4	−16.8	−1.4 [−2.9 to 0.1]	= 0.159	
**6-Min walk test (m)**						
Control	355 ± 104	372 ± 92	4.8	17 [2 to 42]	= 0.307	= 0.008
BW+WB-EMS	401 ± 96	527 ± 127^†^ ^#^	31.3	126 [98 to 154]	= 0.001	
**Handgrip strength (kgf)**						
Control	28.0 ± 7.0	27.7 ± 6.7	−1.1	−0.3 [−1.8 to 1.2]	= 0.303	= 0.022
BW+WB-EMS	30.1 ± 10.7	32.2 ± 10.8^†^ ^#^	7.0	1.1 [0.2 to 2.0]	= 0.004	

*Values expressed in mean ± standard deviation. BW+WB-EMS = body weight combined with electrical muscle stimulation; MD[95% IC] = mean difference and 95% confidence interval*.

† *Significantly greater than the corresponding pre-intervention value (p < 0.05)*.

# *Significantly greater than the control group (p < 0.05)*.

**Figure 3:**
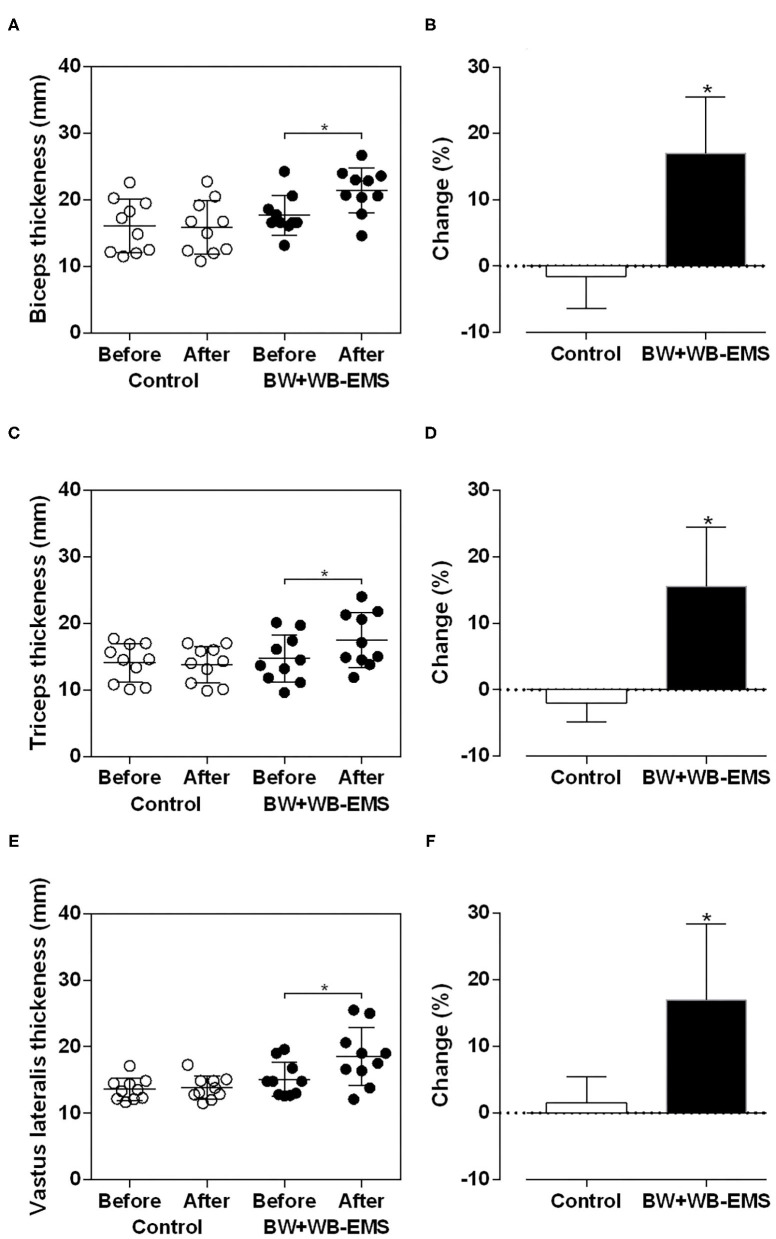
Values expressed as means ± standard deviations of muscle thickness after six weeks of training in control and body weight + whole body electrostimulation (BW + WB − EMS) groups to biceps brachii **(A,B)**, triceps brachii **(C,D)** and vastus lateralis **(E,F)**. *Significantly different from pre-training (*p* < 0.05).

The results of MT are presented in Figure 3. After the intervention there were significant time (*p* = 0.001) and time ^*^ group effects (*p* = 0.001) for the biceps brachii (17.7 ± 3.0 mm vs. 21.4 ± 3.4 mm, Δ% = 21.1; MD [95%CI] = 3.7 [0.3 to 2.5]), triceps brachii (14.7 ± 3.6 mm vs. 17.5 ± 4.1 mm; Δ% = 30.4; MD [95%CI] = 4.5 [1.1 to 4.5]), and vastus lateralis muscles (15.1 ± 2.6 mm vs. 18.6 ± 4.3 mm; Δ% = 29.6; MD [95%CI] = 4.5 [1.7 to 4.3]) in the BW+WB-EMS group. No differences were found in the control group for biceps brachii (16.1 ± 4.0 mm vs. 15.9 ± 4.0 mm, Δ% = −1.4; MD [95%CI] = −0.2 [−0.6 to 1.0]), triceps brachii (14.1 ± 2.9 mm vs. 13.8 ± 2.7 mm; Δ% = −2.0; MD [95%CI] = −0.3 [−0.5 to 0.9]), and vastus lateralis muscles (13.6 ± 1.7 mm vs. 13.8 ± 1.7 mm; Δ% = 1.7; MD [95%CI] = 0.2 [0.3 to 0.9]).

Significant alterations were found in % change (Figure 3) between groups for the biceps brachii (Control: −1.54 ± 4.86% vs. BW+WB-EMS: 17.00 ± 8.47%; *t* = 6.00; MD[95% IC] = 18.55 [12.06 to 25.05]; *p* < 0.001), triceps brachii (Control: −1.94 ± 0.92% vs. BW+WB-EMS: 15.55 ± 2.80%; *t* = 5.91; MD[95% CI] = 17.49 [11.28 to 23.70]; *p* < 0.001), and vastus lateralis muscles (Control: 1.55 ± 1.23% vs. BW+WB-EMS: 16.99 ± 3.82%; *t* = 4.03; MD[95% CI] = 15.44[7.40 to 23.43]; *p* = 0.0008).

No correlations were found in arm curl (Control: *p* = 0.384, *r* = −0.309; 95% CI= −0.78 to 0.39; BW+WB-EMS: *p* = 0.204, *r* = 0.439; 95% CI = −0.26 to 0.83) and handgrip strength (Control: *p* = 0.208, *r* = −0.435; 95% CI = −0.83 to 0.26; BW+WB-EMS: *p* = 0.558, *r* = 0.211; 95% CI = −0.48 to 0.74) with changes in biceps MT. Similar results were found in handgrip strength and triceps MT for control (*p* = 0.598, *r* = −0.190; 95% CI = −0.73 to 0.49) and BW+WB-EMS (*p* = 0.283, *r* = −0.37; 95% CI = −0.81 to 0.33). No correlations were observed in changes in 6-min walk distance (Control: *p* = 0.431, *r* = −0.281; 95% CI = −0.77 to 0.42; BW+WB-EMS: *p* = 0.966, *r* = 0.015; 95% CI = −0.62 to 0.63) and 30-s chair stand test (Control: *p* = 0.257, *r* = −0.395; 95% CI = −0.82 to 0.31; BW+WB-EMS: *p* = 0.187, *r* = 0.433; 95% CI = −0.27 to 0.83) and vastus lateralis MT in either group.

**Figure 4:**
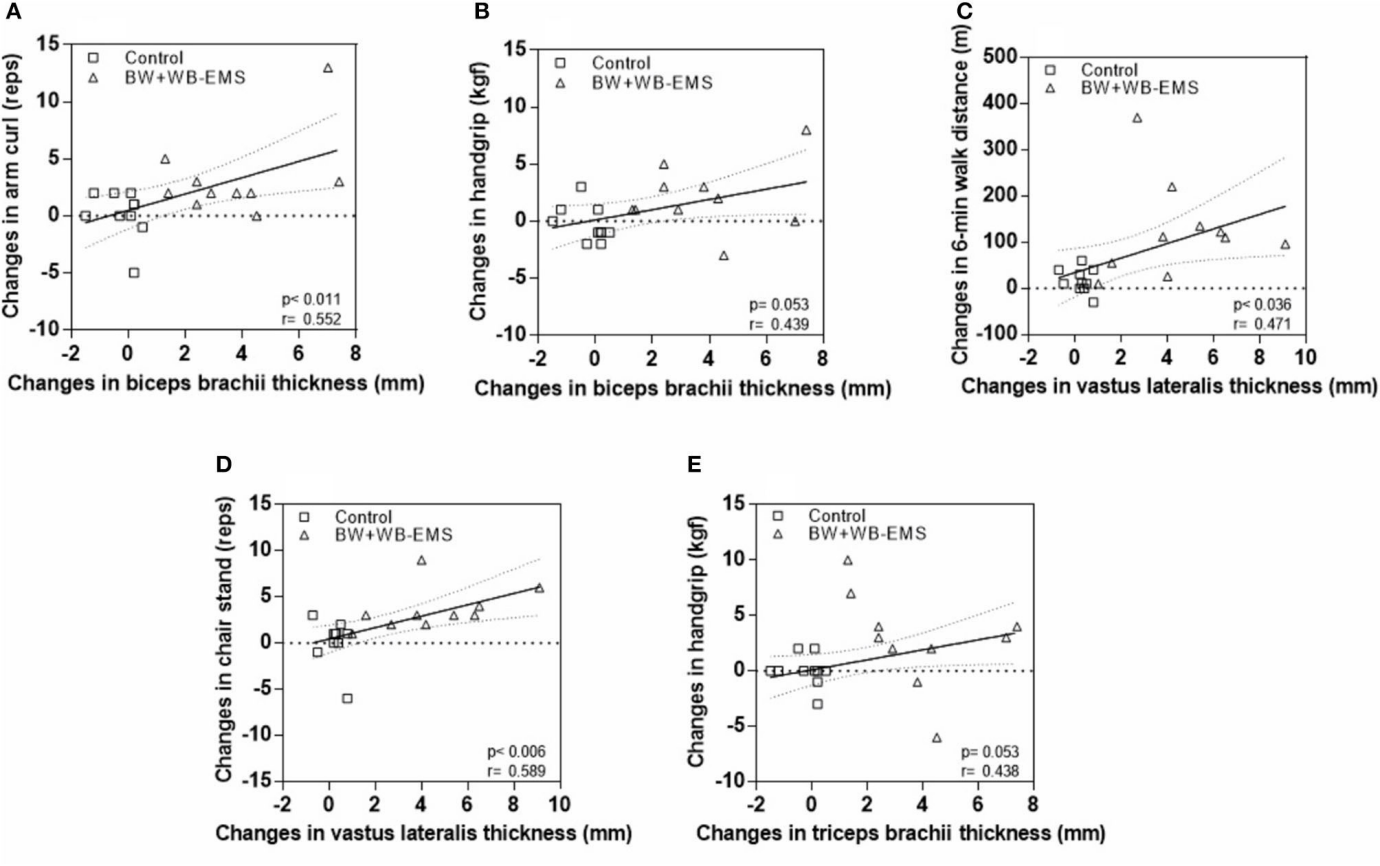
Correlations between changes in functional fitness and muscle thickness parameters. **(A)** Changes in biceps brachii thickness and arm curl. **(B)** Changes in biceps brachii thickness and handgrip. **(C)** Changes in vastus laterais thickness and 6-min walk distance. **(D)** Changes in vastus laterais thickness and chair stand. **(E)** Changes in triceps brachii thickness and handgrip.

Figure 4 presents the general correlations between the changes in performance in functional fitness tests and the changes in MT. Moderate correlations were found in arm curl (*p* = 0.011; *r* = 0.552; 95% CI = 0.14 to 0.79) but not handgrip strength (*p* = 0.053; *r* = 0.439; 95% CC = −0.00 to 0.73) with changes in biceps MT. Moderate changes in 6-min walk distance were significantly correlated with the changes in vastus lateralis MT (*p* = 0.036; *r* = 0.471; 95% CI = 0.03 to 0.75). There was a moderate correlation between the changes in the 30-s chair stand test (*p* = 0.006; *r* = 0.589; 95% CI = 0.19 to 0.81) and the changes in vastus lateralis MT. Furthermore, although a moderate correlation (*r* = 0.438; 95% CI = −0.00 to 0.73) was found between triceps MT and handgrip strength, no significant difference (*p* = 0.053) was reported.

## Discussion

The main results of this study were: (i) BW+WB-EMS increased biceps, triceps, and quadriceps MT; (ii) the BW+WB-EMS group presented increased performance in the 30-s chair stand test, arm curl, 6-min walk test, and handgrip strength test; (iii) there were significant correlations between the changes in functional test performance and the changes in MT.

This is the first study to analyse the effects of WB-EMS on MT in a sample of older men. The results indicated that whole body EMS increased skeletal MT in the upper and lower limbs by up to 20%, indicating that the older subjects presented hypertrophy in the limb muscles after this intervention. These results are in agreement with other studies which used this methodology, demonstrating improvements in muscle mass after a WB-EMS protocol (Benavent-Caballer et al., 2014). Furthermore, it seems that training using WB-EMS is more efficient in generating morphological adaptations in sedentary older men (as is the case of the volunteers in the current study) than in more active older men (Caggiano et al., 1994). Although the mechanisms underlying these effects are not completely understood, increased mechanical stress has been postulated as one of the main stimuli for the process of myofibrillar protein synthesis and consequent muscle hypertrophy (Damas et al., 2019; Evangelista et al., 2019b). In addition, the recruitment of muscle fibers resulting from the WB-EMS may maximize energy expenditure and metabolic stress, stimulating intracellular anabolic and catabolic signaling cascade pathways to increase protein synthesis in the muscle.

We do not find any significant changes in total lean mass after BW+WB-EMS even though an increase in MT was found. However, these data should be interpreted with caution since inconsistencies in the skeletal muscle index obtained by BIA compared with the gold standard have been reported (Buckinx et al., 2015), although this is not an universal finding (Ling et al., 2011).

The use of WB-EMS potentiated the effects of body weight training in the 30-s chair stand, arm curl, 6-min walk, and handgrip strength tests. These results were already expected since electrostimulation activates both type I and type II fibers and even a 4 week-program with 2 or 3 training sessions per week at a frequency between 25 and 50 Hz seems to be enough to trigger positive effects regarding strength and functional tasks (Amiridis et al., 2005; Bezerra et al., 2011). The mechanisms of increases in functional performance were not analyzed, however, the relationship between the changes in MT and the changes in performance in upper and lower limb tests indicates that muscle hypertrophy is a potential mechanism related to improvements in performance (Jones et al., 2016). Vivodtzev et al. (2012) demonstrated a positive association between increases in strength and muscle mass and improved performance in functional tests after 6 weeks of training using electrostimulation in older patients with chronic obstructive pulmonary disease. The authors point out that electrostimulation improved muscle central activation and helped restore the anabolic/catabolic balance in muscle mass, which could have contributed to the gains in muscle strength and hypertrophy and, as a consequence, improved performance in the functional tests.

Severe muscle damage, determined by excessive elevation of Creatine Kinase (CK) and, in more rare cases, the evolution to rhabdomyolysis, are the most common risks associated with WB-EMS reported in the literature (Stöllberger and Finsterer, 2019). However, in the current study, no reports other than mild muscle pain were reported by the volunteers during the study period. It is worth remembering that all sessions were directly supervised by an instructor certified in the use of technology, which increased safety during the training sessions.

Some important limitations in the study should be mentioned. The relatively small sample size and experimental time, no diet/dietary restrictions or energy intake measures, the lack of a gold standard physical test, and previous physical activity level do not allow for further generalizations. Additionally, special attention should be addressed to the large increase in MT in a short training period. Even though ultrasound is a reliable and valid tool for the assessment of muscle size in adults (Evangelista et al., 2019a,b) and older adults (Nijholt et al., 2017; Hida et al., 2018), we measured only one dimension (i.e., MT) of the muscle. In addition, in older adults, the quality of the images obtained is not as good as in a younger population (Ticinesi et al., 2017). For these reasons, the data presented here, as well as the correlation analysis, should be viewed carefully (Zhu, 2012). Thereby, in order to clarify our findings, future research in this area should consider the association between training progression and physically active older people as well as the molecular mechanism and muscle content described.

## Conclusion

Exercise with body weight WB-EMS increases functional capacity and muscle thickness in physically inactive older men. These data support the potential use of WB-EMS to optimize the effects of body weight exercises in the older population.

### Practical Application

Based on the results of the present study, coaches and health professionals should consider the adoption of WB-EMS as an efficient exercise technology in order to promote significant improvements in functional fitness, strength, and muscle mass in older men. Additionally, WB-EMS may represent a time efficient option for those unable or not motivated to join traditional exercise programs.

## Data Availability Statement

The raw data supporting the conclusions of this article will be made available by the authors, without undue reservation.

## Ethics Statement

The studies involving human participants were reviewed and approved by Anhembi Morumbi University (n° 4.028.844/2020). The patients/participants provided their written informed consent to participate in this study.

## Author Contributions

All authors contributed to the preparation of the entire research project, writing, selection of participants and collection of data, data review and analysis, editing, statistical analysis, discussion of results, and execution of the revision.

## Conflict of Interest

AE declares a conflict of interest for working as a scientific adviser for XbodyBrazil. The remaining authors declare that the research was conducted in the absence of any commercial or financial relationships that could be construed as a potential conflict of interest.
