# Corrigendum: Effects of Whole Body Electrostimulation Associated With Body Weight Training on Functional Capacity and Body Composition in Inactive Older People

**DOI:** 10.3389/fphys.2021.694855

**Published:** 2021-05-20

**Authors:** Alexandre Lopes Evangelista, Angelica Castilho Alonso, Raphael M. Ritti-Dias, Bruna Massaroto Barros, Cleison Rodrigues de Souza, Tiago Volpi Braz, Danilo Sales Bocalini, Julia Maria D'andréa Greve

**Affiliations:** ^1^Laboratório de Fisiologia e Bioquímica Experimental, Centro de Educação Física e Esporte, Universidade Federal do Espirito Santo, Vitoria, Brazil; ^2^Programa de Mestrado Ciências do Envelhecimento, Universidade São Judas Tadeu, São Paulo, Brazil; ^3^Programa de Pós-graduação em ciências da reabilitação, Universidade Nove de Julho, São Paulo, Brazil; ^4^Laboratório de Avaliação do Movimento Humano, Universidade Metodista de Piracicaba, Piracicaba, Brazil; ^5^Departamento de Ortopedia e Traumatologia, Universidade de São Paulo Faculdade de Medicina, São Paulo, Brazil

**Keywords:** older adults, electrostimulation, physical function, body composition, functional fitness

In the original article, there was a mistake in the legend for ^**^Table 2^**^ as published. The incorrect legend reads as “^****^ ST+EMS= strength training combined with electrical muscle stimulation.” The correct legend should read as 2 “BW+WB-EMS: body weight associated with whole body electrostimulation^****^.” The correct legend appears below.

In the original article, there was a mistake in ^**^Table 2^**^. ^**^The lean mass signs †# need to be removed, since there was no statistical difference between pre *versus* post^**^. The corrected Table 2 appears below.

**Table 2:** Alterations on body composition and functional fitness after 6 weeks of strength training combined with electrical muscle stimulation.

**Parameters**	**Pre**	**Post**	**Δ%**	**MD [95%CI]**	**Time**	Time*Group
					***p*-value**	***p*-value**
**BODY COMPOSITION**						
**Body mass (kg)**						
Control	69.9 ± 11.7	67.6 ± 11.9	0.5	2.3 [−1.2 to 2.8]	= 0.402	= 0.507
BW+WB-EMS	76.2 ± 16.2	76.2 ± 16.9	−0.6	−0.1 [2.2 to 5.4]	= 0.504	
**Fat body (%)**						
Control	31.8 ± 12.2	31.8 ± 12.7	−0.1	0.1 [−0.7 to 0.9]	= 0.672	= 0.534
BW+WB-EMS	34.6 ± 6.6	35.0 ± 7.1	1.0	0.4 [−0.5 to 2.5]	= 0.388	
**Lean mass (kg)**						
Control	45.7 ± 8.6	45.6 ± 8.2	−0.2	−0.1 [−0.4 to 0.2]	= 0.409	= 0.438
BW+WB-EMS	49.4 ± 12.1	50.0 ± 11.1	1.1	0.6 [−0.3 to 1.5]	= 0.327	
**FUNCTIONAL FITNESS**						
**Sitting-rising test (reps)**						
Control	11.8 ± 4.9	12.0 ± 2.7	1.7	0.2 [−0.1 to 0.5]	= 0.192	= 0.024
BW+WB-EMS	10.2 ± 3.3	13.8 ± 5.0^†^ ^#^	35.3	2.6 [1.3 to 3.9]	= 0.022	
**Arm curl (reps)**						
Control	14.3 ± 3.2	14.5 ± 2.9	1.4	0.2 [−0.8 to 1.2]	= 0.289	= 0.012
BW+WB-EMS	16.6 ± 3.9	19.9 ± 6.1^†^ ^#^	19.9	3.3 [0.9 to 5.7]	= 0.007	
**Stationary march test (reps)**						
Control	36.8 ± 11.4	37.4 ± 9.2	1.6	0.8 [−0.4 to 2.0]	= 0.289	= 0.045
BW+WB-EMS	51.2 ± 23.8^#^	52.5 ± 19.0^#^	2.5	1.3 [0.1 to 2.5]	= 0.183	
**Back scratch test-left (cm)**						
Control	19.0 ± 16.1	18.5 ± 15.2	−2.7	−0.5 [−2.1 to 1.1]	= 0.128	= 0.023
BW+WB-EMS	9.1 ± 11.1^#^	9.5 ± 8.3^#^	4.4	0.4 [−0.7 to 1.5]	= 0.107	
**Back scratch test-right (cm)**						
Control	16.4 ± 13.9	15.0 ± 12.5	−8.4	−1.4 [−3.6 to 0.8]	= 0.338	= 0.042
BW+WB-EMS	7.0 ± 8.5^#^	5.1 ± 7.0^#^	−27.1	−1.9 [−3.9 to 0.1]	= 0.256	
**8 feet up-and-go (s)**						
Control	10.5 ± 3.3	9.4 ± 3.0	−10.7	−1.1 [−3.6 to 1.4]	= 0.202	= 0.132
BW+WB-EMS	8.6 ± 3.0	7.2 ± 2.4	−16.8	−1.4 [−2.9 to 0.1]	= 0.159	
**6-Min walk test (m)**						
Control	355 ± 104	372 ± 92	4.8	17 [2 to 42]	= 0.307	= 0.008
BW+WB-EMS	401 ± 96	527 ± 127^†^ ^#^	31.3	126 [98 to 154]	= 0.001	
**Handgrip strength (kgf)**						
Control	28.0 ± 7.0	27.7 ± 6.7	−1.1	−0.3 [−1.8 to 1.2]	= 0.303	= 0.022
BW+WB-EMS	30.1 ± 10.7	32.2 ± 10.8^†^ ^#^	7.0	1.1 [0.2 to 2.0]	= 0.004	

*Values expressed in mean ± standard deviation. BW+WB-EMS = body weight combined with electrical muscle stimulation; MD[95% IC] = mean difference and 95% confidence interval*.

† *Significantly greater than the corresponding pre-intervention value (p < 0.05)*.

# *Significantly greater than the control group (p < 0.05)*.

In the original article, there was an error in the first paragraph of the ^**^Results^**^ section It currently reads as “No differences were found in baseline parameters for any outcome parameters. As presented in Table 2, the values of sitting-rising test, arm curl, 6-min walk test, and handgrip strength were different from the pre-intervention value and the control group. However, the values in the stationary march test, and back scratch test, left and right side, were different only for the control group. No differences were found in the 8 feet up-and-go test.”

The paragraph should read as “As presented in Table 2, significant differences were found in baseline parameters between the control group and the BW+WB-EMS group for the stationary march test and the Back scratch test. The values of sitting-rising test, arm curl, 6-min walk test, and handgrip strength were different from the pre-intervention value and the control group. However, the values in the stationary march test, and back scratch test, left and right side, were different only for the control group. No differences were found in the 8 feet up-and-go test.”

The authors apologize for this error and state that this does not change the scientific conclusions of the article in any way. The original article has been updated.

