# Corrigendum: Effects of Whole Body Electrostimulation Associated With Body Weight Training on Functional Capacity and Body Composition in Inactive Older People

**DOI:** 10.3389/fphys.2021.714782

**Published:** 2021-07-23

**Authors:** Alexandre Lopes Evangelista, Angelica Castilho Alonso, Raphael M. Ritti-Dias, Bruna Massaroto Barros, Cleison Rodrigues de Souza, Tiago Volpi Braz, Danilo Sales Bocalini, Julia Maria D'andréa Greve

**Affiliations:** ^1^Laboratório de Fisiologia e Bioquímica Experimental, Centro de Educação Física e Esporte, Universidade Federal Do Espirito Santo, Vitoria, Brazil; ^2^Programa de Mestrado Ciências Do Envelhecimento, Universidade São Judas Tadeu, São Paulo, Brazil; ^3^Programa de Pós-Graduação em Ciências da Reabilitação, Universidade Nove de Julho, São Paulo, Brazil; ^4^Laboratório de Avaliação Do Movimento Humano, Universidade Metodista de Piracicaba, Piracicaba, Brazil; ^5^Departamento de Ortopedia e Traumatologia, Universidade de São Paulo Faculdade de Medicina, São Paulo, Brazil

**Keywords:** older adults, electrostimulation, physical function, body composition, functional fitness

In the original article, there was a mistake in Figure 4 as published. The axes (X and Y) are inverted. The corrected Figure 4 appears below.

**Figure 4:**
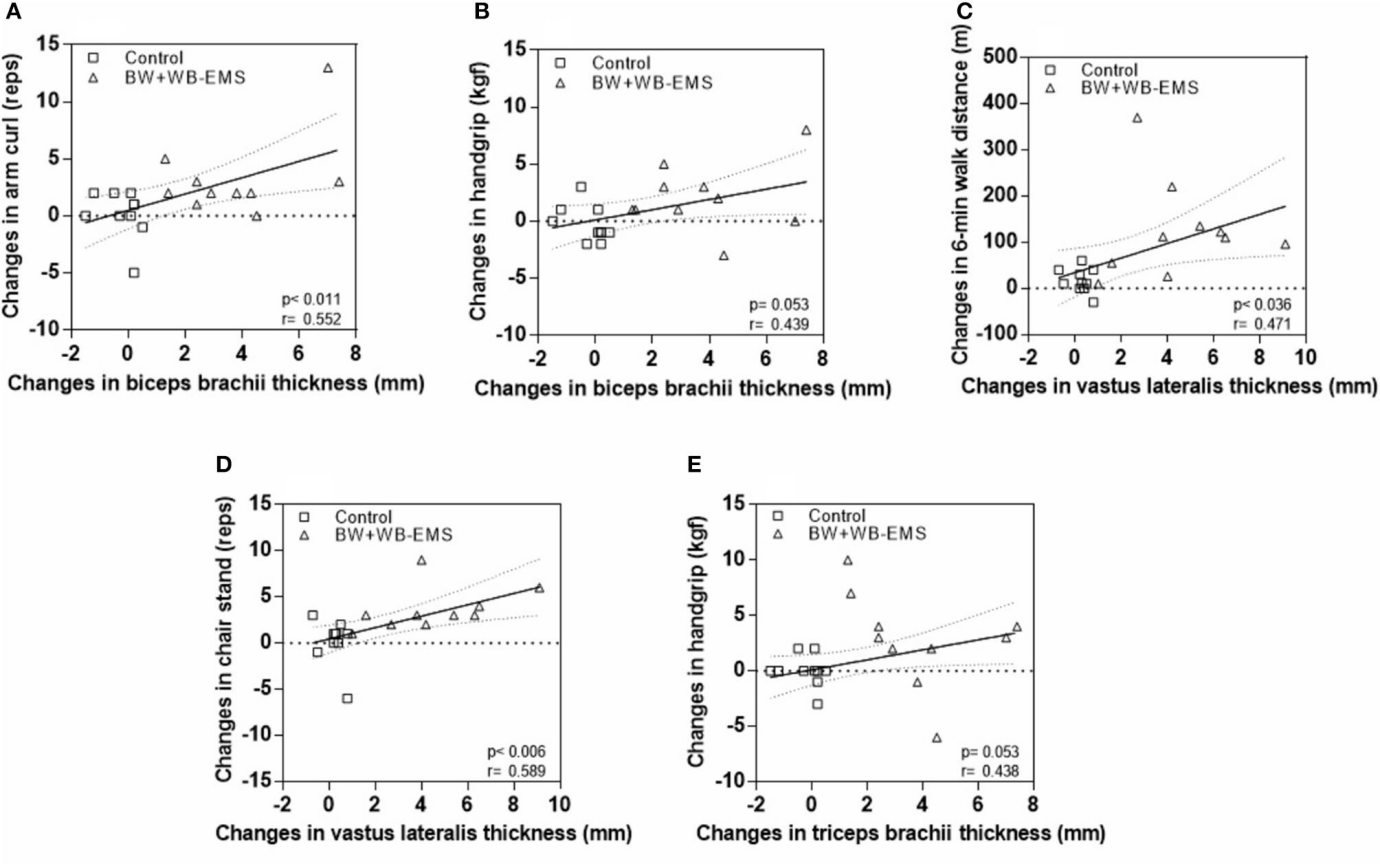
Correlations between changes in functional fitness and muscle thickness parameters. **(A)** Changes in biceps brachii thickness and arm curl. **(B)** Changes in biceps brachii thickness and handgrip. **(C)** Changes in vastus laterais thickness and 6-min walk distance. **(D)** Changes in vastus laterais thickness and chair stand. **(E)** Changes in triceps brachii thickness and handgrip.

In the original article, there was an error. The age of the participants was not indicated.

A correction has been made to METHODS, Subjects, Paragraph Number 3 (at the end):

All participants (30 subjects) were then assigned to either the body weight associated with whole body electrostimulation group (BW+WB-EMS) or control group using a computerized random-number generator. The randomization process occurred in blocks of five subjects. Each block resulted in the allocation of two subjects to the BW+WB-EMS and two subjects to the control group, ensuring a recruitment balance of 1:1 throughout the study. Both groups performed the same exercise protocol with body weight, however the BW+WB-EMS group (*n* = 15) performed the exercises with whole body electrostimulation, while the control group (*n* = 15) carried out the exercises without receiving electrical current stimuli. During follow up, three subjects in the BW+WB-EMS group and four in the control group dropped out of the study. Thus, 20 subjects (mean age 75.1± 6.58 years) were analyzed in this study, as shown in Figure 1.

The authors apologize for this error and state that this does not change the scientific conclusions of the article in any way. The original article has been updated.

## Publisher's Note

All claims expressed in this article are solely those of the authors and do not necessarily represent those of their affiliated organizations, or those of the publisher, the editors and the reviewers. Any product that may be evaluated in this article, or claim that may be made by its manufacturer, is not guaranteed or endorsed by the publisher.

